# Supplementary material for: Fertility and Pregnancy Outcomes after Fertility-Sparing Surgery for Early-Stage Borderline Ovarian Tumors and Epithelial Ovarian Cancer: A Single-Center Study
Source: Cancers (Basel). 2023 Nov 8;15(22):5327. doi: 10.3390/cancers15225327 (PMC10670285; doi:10.3390/cancers15225327)
Supplement: Supplementary file 1 [file cancers-15-05327-s001.zip › cancers-2630481-supplementary.pdf]

Supplementary Tables

Supplementary Table S1. Analysis of risk factors for menstruation irregularity in patients with epithelial ovarian cancer

	Menstrual Irregularity (%)				*p* value
FIGO stage	Ia	Ib		Ic	-
	13.33 (2/15)	-		13.33 (2/18)	
Comprehensiveness of FSS	sFSS		cFSS		0.9340
	15.38 (2/13)		10 (2/20)		
Ovary part of FSS	Salpingo-oophorectomy only		Cystectomy-included		-
	13.79 (4/29)		0 (0/4)		
Underlying medical disease	Endocrine	Autoimmune		No	0.0977
	0 (0/1)	50 (2/4)		7.14 (2/28)	
BMI	<18.5	18.5-25		>25	0.8221
	0 (0/4)	13.64 (3/22)		14.28(1/7)	
Prior menstrual regularity	Regular		Irregular		0.1422
	7.41 (2/27)		33.33 (2/6)		
Adjuvant chemotherapy	Yes		No		0.9341
	7.69 (1/13)		15 (3/20)		
Histology	Mucinous	Clear cell	Serous	Endometrioid	-
	13.33 (2/15)	10 (1/10)	0 (0/1)	14.29 (1/7)	
Prior GYN OP	Yes		No		-
	0 (0/4)		13.79 (4/29)		

Supplementary Table S2. Analysis of risk factors for menstruation irregularity in patients with borderline ovarian tumors

	Menstrual Irregularity (%)			*p* value
FIGO stage	Ia	Ib	Ic	0.2409
	0 (0/38)	0 (0/2)	6.45 (2/31)	
Histology	Mucinous	Serous	Seromucinous	0.4601
	1.92 (1/52)	0 (0/4)	0 (0/15)	
Prior GYN OP	Yes	No		0.2604
	10 (1/10)	1.64 (1/61)		
Comprehensiveness of FSS	sFSS	cFSS		0.6936
	2.27 (1/44)	3.7 (1/27)		
Ovary part of FSS	Salpingo-oophorectomy only	Cystectomy-included		0.4455
	1.89 (1/53)	5.56 (1/18)		
Underlying medical disease	Endocrine	Autoimmune	No	-
	0 (0/2)	0 (0/1)	2.94 (2/68)	
BMI	<18.5	18.5-25	>25	-
	0 (0/5)	4.08 (2/49)	0 (0/17)	
Prior menstrual regularity	Regular	Irregular		-
	2.86 (2/70)	0 (0/1)		
Adjuvant chemotherapy	Yes	No		-
	0 (0/3)	2.94 (2/68)		

Supplementary Table S3. Analysis of risk factors for recurrence in patients with epithelial ovarian cancer

	Recurrence (%)				*p* value
FIGO stage	Ia	Ib		Ic	-
	0 (0/15)	-		5.56 (1/18)	
Histology	Mucinous	Clear cell	Serous	Endometrioid	0.5466
	0 (0/15)	10 (1/10)	0 (0/1)	0 (0/7)	
Preoperative CA-125	<35U/ml	35-100U/ml		>100U/ml	0.5163
	0 (0/16)	9.09 (1/11)		0 (0/6)	
Comprehensiveness of FSS	sFSS		cFSS		-
	0 (0/13)		5 (1/20)		
Ovary part of FSS	Salpingo-oophorectomy only		Cystectomy-included		-
	3.45 (1/29)		0 (0/4)		
Lymphadenectomy	Yes		No		-
	5.56 (1/18)		0 (0/15)		
Omentectomy	Yes		No		-
	4.76 (1/21))		0 (0/12)		
Adjuvant chemotherapy	Yes		No		-
	5 (1/20)		0 (0/13)		
